# Choline-Containing Phospholipids in Stroke Treatment: A Systematic Review and Meta-Analysis

**DOI:** 10.3390/jcm12082875

**Published:** 2023-04-14

**Authors:** Getu Gamo Sagaro, Francesco Amenta

**Affiliations:** Clinical Research, Telemedicine and Telepharmacy Center, School of Medicinal and Health Products Sciences, University of Camerino, 62032 Camerino, Italy

**Keywords:** citicoline, CDP-choline, cytidine-5-diphosphatidylcholine, choline alphoscerate, alpha-glyceryl-phosphorylcholine, α-GPC, stroke

## Abstract

Background: Globally, stroke is the second leading cause of death and disability. In different studies conducted previously, the choline-containing phospholipids citicoline and choline alphoscerate have been proposed as adjuvants in the treatment of acute strokes. A systematic review was conducted to provide updated information on the effects of citicoline and choline alphoscerate in patients with acute and hemorrhagic strokes. Methods: PubMed/Medline, Scopus, and Web of Science were searched to identify relevant materials. Data were pooled, and odds ratios (OR) were reported for binary outcomes. Using mean differences (MD), we evaluated continuous outcomes. Results: A total of 1460 studies were reviewed; 15 studies with 8357 subjects met the eligibility criteria and were included in the analysis. In our study, citicoline treatment did not result in improved neurological function (NIHSS < 1, OR = 1.05; 95% confidence interval (CI): 0.87–1.27) or functional recovery (mRS < 1, OR = 1.36; 95% CI: 0.99–1.87) in patients with acute stroke. Choline alphoscerate improved neurological function and functional recovery in stroke patients based on the Mathew’s scale and the Mini-Mental State Examination (MMSE). Conclusion: Citicoline did not improve the neurological or functional outcomes in acute stroke patients. In contrast, choline alphoscerate improved neurological function and functional recovery and reduced dependency in stroke patients.

## 1. Introduction

Strokes are the second leading cause of death and disability worldwide. It is estimated that one in four people will suffer a stroke during their lifetime [1]. Each year, 15 million people worldwide suffer from strokes. Of these, 5 million die, while another 5 million become permanently disabled, placing an enormous burden on families and communities [2]. According to a recent study published in the Lancet, stroke-related deaths are expected to increase from 6 million in 2010 to 12 million by 2030 [3].

Several clinical trials have been conducted in order to identify effective treatments for stroke, which remains a medical emergency owing to the potential for severe disability [4,5,6]. Choline-containing phospholipids, which are acetylcholine precursors such as citicoline and choline alphoscerate, have been proposed as adjuvants in the treatment of acute strokes. In patients with acute stroke, choline-containing phospholipids play an important role. The choline-containing phospholipids citicoline (cytidine 5′-diphosphocholine or CDP-choline) and choline alphoscerate (alpha-glyceryl-phosphorylcholine or α-GPC) are precursors of acetylcholine (Ach) [7]. These compounds are used as a substrate for acetylcholine synthesis, which can improve cholinergic neurotransmission [7]. The precursors of Ach can also be used in neuroprotective substances as well as in neurogenesis after an ischemic stroke [7,8]. It appears that choline supplementation at each stage of brain development enhances the performance of the brain, perhaps due to membrane synthesis during neuronal development; therefore, it requires a sufficient supply to maintain proper brain function. It plays a critical role in a variety of neurochemical pathways [8].

Choline-containing phospholipids may have anti-inflammatory effects [8]. As a result of their anti-inflammatory effects, citicoline and choline alphoscerate may be beneficial to the microglia, which play an important role in the resolution of local inflammation, the removal of cellular debris, and the provision of protective factors in ischemic brains to reduce cell injury [7,8,9,10]. Recent research has implicated inflammation in the pathological abnormalities that accompany stroke, specifically the tryptophan–serotonin–kynurenine axis, which mediates the neurotoxic effects of inflammation [11]. Thus, choline-containing phospholipids, and in particular choline alphoscerate, may have anti-inflammatory properties that may explain their neuroprotective effects following stroke and the associated functional improvement. Citicoline is an essential precursor of phosphatidylcholine, a structural component of cell membranes that is degraded to free fatty acids and free radicals during cerebral ischemia. In terms of choline alphoscerate, various studies have been conducted to determine if it enhances cognitive function and functional outcomes in patients suffering from acute cerebrovascular diseases (such as strokes and transient ischemic attacks (TIAs)) [12,13].

Previous studies have demonstrated that citicoline has an excellent safety profile when treating acute ischemic stroke patients [14,15]. An analysis of pooled patient data from citicoline trials in acute and subacute stroke patients revealed a substantial and beneficial effect, with absolute reductions of 10–12% in the risks of long-term mortality and disability [16]. In another study, the odds ratio (OR) of complete neurological and functional recovery in acute ischemic stroke patients treated with citicoline was 1.33 (1.10–1.62) [17]. Lee M. et al. [18] (2010) found that citicoline treatment reduced mortality or dependency in acute stroke patients (OR = 0.65, 95% CI: 0.54–0.77). Different clinical trials have reported conflicting results regarding the effects of citicoline on patients with acute strokes. A clinical trial conducted in the United States of America (USA) on 899 patients with acute ischemic stroke reported that citicoline was ineffective in improving neurological and functional outcomes at the end of the scheduled follow-up period [5]. Recently, a randomized controlled trial failed to prove the benefit of citicoline treatment in neurological and functional recovery from an acute ischemic stroke in 90 days [4]. In many European countries, citicoline is used for treating cognitive impairment, particularly in cases involving predominantly cerebrovascular disease [19].

The purpose of this systematic review was to provide updated information regarding the effects of the choline-containing phospholipids citicoline and choline alphoscerate in patients with acute and hemorrhagic strokes. A second objective was to assess the treatment effects of citicoline and choline alphoscerate in patients with acute strokes in a comparative manner. In this study, we performed a meta-analysis to evaluate the treatment effects of both citicoline and choline alphoscerate on the outcome of stroke patients by using the National Institutes of Health Stroke Scale (NIHSS < 1), the modified Rankin Scale (mRS < 1), the Mini-Mental State Examination (MMSE > 23), and the Barthel Index (BI > 95) scale.

## 2. Materials and Methods

The present systematic review followed the Preferred Items for Systematic Review and Meta-analysis (PRISMA) checklists and diagrams to design and report the results [20]. A protocol for this systematic review has been registered with the International Prospective Register of Systematic Reviews (PROSPERO), and the registration number is CRD42023401396. It is available at: https://www.crd.york.ac.uk/prospero/display_record.php?ID=CRD42023401396 (accessed on 4 March 2023).

### 2.1. Search Strategy and Data Sources

A comprehensive systematic search was conducted using international databases such as PubMed, Web of Science (WOS), and Embase in order to identify relevant studies. The reference lists of the retrieved studies were manually reviewed for further relevant articles. To combine the search terms for each outcome of interest, we used Boolean operators such as “AND” and “OR”. PubMed, Embase, and WOS were searched using the following key terms: (1) Citicoline OR cytidine diphosphate choline OR CDP-choline OR cytidine 5’-diphosphocholine OR citocholine OR cyticholine OR cytidine-5-diphosphocholine; (2) L-Alpha glycerylphosphorylcholine OR alpha-GPC OR choline alphoscerate OR Cereton or α-Glycerylphosphorylcholine OR alpha-glyceryl-phosphorylcholine OR a-GPC; (3) acute ischaemic stroke OR acute ischemic stroke OR acute ischaemic OR acute ischemic OR cerebrovascular diseases OR brain ischemia OR brain ischaemia OR cerebrovascular disorders; (4) hemorrhagic stroke OR brain haemorrhage OR cerebrovascular accident OR intracerebral haemorrhage OR subarachnoid haemorrhage OR cerebral haemorrhage OR cerebrovascular insult; (5) 1 AND 3; (6) 1 AND 4; (7) 2 AND 3; (8) 2 AND 4; (9) limit 5, 6, 7, 8 to humans. The full search strategy in PubMed for acute and hemorrhagic stroke can be found in the supplementary file (see Supplementary Table S1).

### 2.2. Inclusion and Exclusion Criteria

Our study included randomized control trials (RCTs), case-control studies, and cohort studies with and without control groups. This systematic review evaluated studies that enrolled patients with acute ischemic stroke or hemorrhagic stroke and assessed neurological function, functional recovery, and independence using the National Institutes of Health Stroke Scale (NIHSS), modified Rankin Scale (mRS), and Barthel Index (BI). Patients suffering from other conditions, such as dementia and Alzheimer’s disease, were excluded from the study. Furthermore, studies comparing cholinergic precursors (citicoline and choline alphoscerate) to placebo or any other active treatment were also included. Our study did not place any restrictions on the route of administration, the dose, the duration of treatment, or the publication date. We excluded studies with unclear methods involving NIHSS, mRS, or BI and studies published only in conference proceedings. 

### 2.3. Selection of Studies

The title and abstract were screened based on eligibility criteria by two independent reviewers. We retrieved full-text articles from the databases following the screening of titles and abstracts, and two reviewer authors independently reviewed the full-text articles to select studies that met our inclusion criteria. Any disagreements regarding article selection were resolved through discussion.

### 2.4. Data Extraction and Management

After studies were selected based on inclusion criteria, data were independently extracted. Using an Excel spreadsheet, the review authors extracted data, including first author’s name, study design, year of publication, number of patients with acute ischemic stroke or hemorrhagic stroke, active treatment with dose, route of administration, treatment duration, test scales used, type of control, and NIHSS score at baseline if available. 

### 2.5. Outcome Measures

The primary objective of this systematic review was to determine the effects of citicoline and choline alphoscerate on patient recovery using NIHSS, BI, and mRS/MMAS after the end of the scheduled follow-up period for stroke (acute or hemorrhagic) patients. The pooling of patient data for assessing neurological function, functional recovery, and activity of daily living outcomes was conducted using the NIHSS, mRS/MMSE, or BI scores, and the treatment effect on each of the scales was evaluated by odds ratio (OR) if a binary outcome was available. Secondary outcome was to compare the treatment effects of choline alphoscerate with citicoline on neurological function and functional outcome in stroke patients.

### 2.6. Quality Assessment

The methodological quality of RCT studies was evaluated using the Cochrane Collaboration tool [21,22]. As a part of the assessment of the selected RCTs, we considered six domains, including the method of randomization, concealment of allocation, blinding of investigators and patients, blinding of outcome assessment, adequate follow-up, and other possible biases. A score of “yes,” “no,” or “unclear” was given to each of the domains. Based on the quality assessment, studies were divided into three categories: low-risk bias, moderate-risk bias, and high-risk bias. For nonrandomized studies, we used the Newcastle–Ottawa Scale [23] and scored each study on 9 items and assigned a maximum of 9 points in 3 areas, including selection, comparability, and the outcomes of interest. Therefore, we evaluated the quality of the selected studies based on the agreed-upon category scores ranging from 0 to 9 and categorized them into three groups: low quality (0–4), moderate quality (5–6), and high quality (7–9).

### 2.7. Statistical Analysis

For binary outcomes, such as neurological function and functional recovery as measured by NIHSS, mRS, and BI scales, the odds ratio (OR) was calculated if available. The mean differences and standard deviations were used for continuous outcomes. Three endpoints (neurological function, functional recovery, and independence or improvement in activities of daily living) were assessed using the National Institutes of Health Stroke Scale (NIHSS < 1), modified Rankin Scale (mRS < 1) or MMSE, and Barthel Index (BI > 95), respectively, and we estimated ORs with 95% confidence intervals (CIs) on each scale of the outcome when compared with control groups by random effect models. 

Heterogeneity between studies was assessed using the Cochran’s Q test [24] and *I*^2^ test statistics [25]. The degree of heterogeneity was considered low, moderate, or high based on *I*^2^ values of less than 25%, 25% to 75%, and more than 75%, respectively [26]. The data were entered into a Microsoft Excel spreadsheet and analyzed using R-software (Version 4.1.1, The R Foundation for Statistical Computing, Vienna, Austria) [27]. We used R metafor package [28] along with different arguments to calculate individual effect sizes.

## 3. Results

### 3.1. Relevant Articles

**Figure 1:**
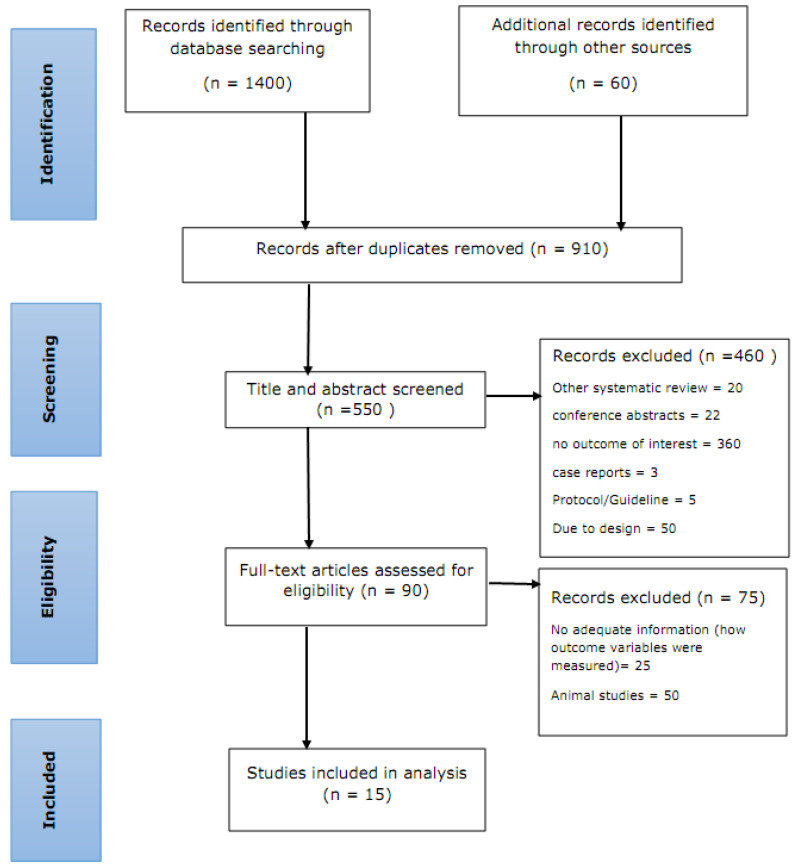
Flowchart illustrating the process of study search, selection, and inclusion in this systematic review and meta-analysis using PRISMA 2020 [29].

After searching the databases, we found a total of 1460 records, of which 810 were excluded because of duplication. During the title and abstract screening, 560 records were excluded. The remaining 90 full-text articles were then evaluated. There were 90 full-text papers reviewed, of which 50 animal studies and 25 human studies did not meet the eligibility criteria. Finally, 15 studies (13 on acute ischemic stroke and 2 on hemorrhagic stroke) were included as part of this systematic review. Figure 1 shows the entire process of finding, selecting, and including the studies. 

The studies included in this systematic review and meta-analysis were conducted up to May 2022. Among the included studies, eleven were randomized controlled trials, three were uncontrolled trials, and one was a case-control study. Table 1 summarizes the characteristics of the selected studies.

### 3.2. Methodological Quality Assessment

The methodological quality of the studies included in this systematic review was evaluated using both the Cochrane Collaboration tool for RCTs and the Newcastle–Ottawa Scale tool for non-RCTs. As a result, 81.8% (*n* = 9) of the randomized controlled trials showed low-risk bias, while 18.2% (*n* = 2) demonstrated moderate-risk bias (Supplementary Table S2). On the other hand, all nonrandomized controlled trials (*n* = 4) were found to be of high methodological quality (see Supplementary Table S3). 

### 3.3. Effect of Citicoline on Stroke Patients

**Figure 2:**
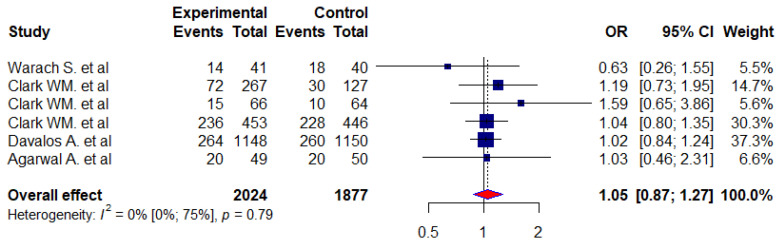
The forest plot of treatment effect of citicoline on neurological function (using NIHSS < 1).

The citicoline effect on neurological function was evaluated using NIHSS scale scores in six randomized controlled trial (RCT) studies [4,5,30,32,33,34]. In these 6 studies, a total of 3901 patients with acute ischemic stroke were randomized into 2 groups: citicoline (*n* = 2024) and placebo (*n* = 1877). A total of 621 (31%) out of 2024 experimental patients and 30% (566) out of 1877 control patients showed NIHSS < 1. We found an odds ratio (OR) of 1.05 with 95% confidence intervals (0.87–1.27) between the experimental and control groups (Figure 2).

**Figure 3:**
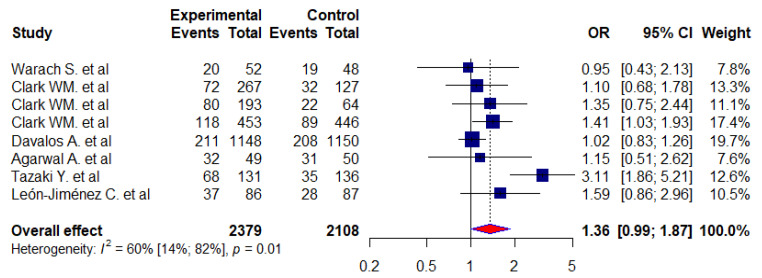
The forest plot of treatment effect of citicoline on functional recovery (using mRS < 1).

We used the modified Rankin Scale (mRS < 1) to evaluate the functional recovery following a stroke. Our analysis consisted of 8 studies [4,5,30,31,32,33,34,35] (7 RCTs and 1 case control) with 4487 stroke patients, and our aim was to evaluate the functional recovery following a stroke using the modified Rankin Scale (mRS < 1). A total of 27% and 22% of the experimental and control patients, respectively, showed an mRS score of <1. A meta-analysis was performed, and the odds ratio was calculated as 1.36 (0.99–1.87). We also found that there was statistically significant heterogeneity between the included studies (I^2^ = 60%, *p* < 0.05) (Figure 3).

**Figure 4:**
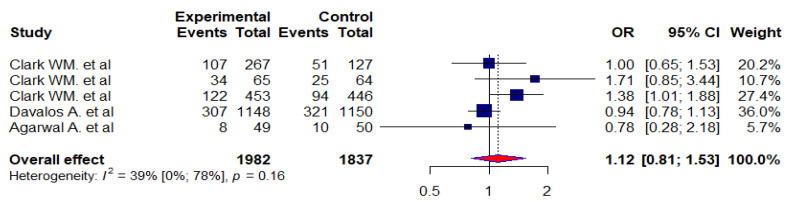
The forest plot of treatment effect of citicoline on outcomes of activities of daily living for patients with acute stroke (using BI > 95).

As part of the assessment of their activities of daily living, acute stroke patients were assessed using the Barthel Index (BI). Overall, 5 studies [4,5,30,32,34] were identified with a total of 3819 study participants in 2 groups (the experimental group (*n* = 1982) and the control group (*n* = 1837)). In general, there was an improvement in 29.2% of the patients in the experimental group and 27.3% of the patients in the control group (BI > 95). Nevertheless, we found a 1.12 odds ratio (0.81–1.53) between the experimental and control groups and a 39% level of heterogeneity among all included studies (Figure 4).

We also analyzed two studies to examine the effects of citicoline on functional recovery in patients with hemorrhagic strokes following treatment. Generally, 2 randomized control trials [36,37] with a total of 1055 study participants in 2 groups were identified. In the experimental group, 527 participants were included, while in the control group, 528 study subjects were included. Secades JJ and his colleagues compared the functional recovery of the two groups (experimental and control) using a modified Rankin Scale (mRS < 1). A second study was conducted using the Glasgow Coma Scale (GCS) to determine the functional status of patients with hemorrhagic stroke. By performing a meta-analysis, we determined that the odds ratio between the two groups was 1.75 (0.00–964.00), with an insignificant heterogeneity between the included studies (I^2^ = 61%, *p*-value = 0.11).

### 3.4. Effect of Choline Alphoscerate on Patients with Stroke

As a part of our literature review, we identified 5 [6,38,39,40,41] clinical trials that included 2544 patients with acute stroke. The clinical assessments were conducted in four trials in two phases: In the first phase, patients were given choline alphoscerate parenterally for one month (three trials) [6,39,40], and their functional recovery was determined according to Mathew’s scale. As part of the second phase, from the second month to six months following stroke onset, the patients in three studies were orally treated with α-Glycerophosphocholine (choline alphoscerate), and the efficacy of the treatment was tested using the Mini-Mental State Examination (MMSE), the Global Deterioration Scale (GDS), or the Crichton Geriatric Rating Scale (CGRS). However, we used the MMSE test to assess the effectiveness of choline alphoscerate in the second phase, calculating the mean difference between the end of the scheduled follow-up period (the sixth month) and the end of the second month. In other words, in three of the studies included, the neurological and functional outcomes of the patients with stroke were evaluated using Mathew’s scale and the MMSE test [6,39,40]. The NIHSS and mRS scores were used in two studies out of five to evaluate the effects of choline alphoscerate treatment on neurological and functional outcomes [38,41].

The Mini-Mental State Examination (MMSE) [42] is a validated screening instrument that is used as a general measure of cognitive recovery among patients with stroke. It is a test that evaluates patients based on 11 items and analyzes the patient’s abilities in orientation, attention, language, calculation, memory fixation, and constructive practice. In general, the lower the score, the greater the impairment, and the results of the test are presented in a range between 0 and 30. As a result, the lower limit of the ‘normal range’ of cognitive functions is 23. Mathew’s scale [43] is used to measure the degree of impairment following a stroke. It was developed to assess the functional status of patients following an acute stroke. Mathew’s scale analyzes both cognitive and neurological factors, including language, cranial nerve function, motor function, and sensory function.

In three clinical trials [6,39,40] evaluating the effect of choline alphoscerate in treating stroke patients, there was no control group with either a placebo or other active drugs. Consequently, the investigators assessed the efficacy of the treatment on different individual scales by calculating the means and standard deviations at a baseline level and the first month, second month, third month, and sixth month following the stroke. As a result, the outcome is continuous; that is, the outcome for each study was a summarized mean with a standard deviation. In order to assess the efficacy of choline alphoscerate on the neurological and functional outcomes in stroke patients, we evaluated the mean difference between both groups. The mean difference (MD) was calculated by computing the difference between the baseline mean and the mean on the 28th day of the first month or the first month in the first phase, and between the mean for the second month and that at the end of the scheduled date (6 months) in the second phase, along with the *p*-value for each study. Accordingly, a positive score for the mean difference indicated an improvement in the treatment effect. 

### 3.5. Effect of Citicoline and Choline Alphoscerate on Acute Stroke Patients

We assessed the effects of citicoline on neurological function, functional recovery, and independence or improvement in daily living function of patients with acute stroke using the NIHSS, mRS, and BI scale scores and compared the experimental groups with the controls. We found no significant difference between the experimental and control groups in neurological function on the NIHSS scores based on the meta-analysis (OR = 1.05, 95% CI: 0.87 to 1.27). On the functional outcome, we performed a meta-analysis of eight studies where we evaluated the functional recovery of stroke patients using a score calculated from the mRS scale. Therefore, our odds ratio (OR) suggests that citicoline is similar to a placebo for mRS improvement when compared to the placebo group. In other words, our pooled patient data analysis indicated that citicoline did not have a significant effect on the functional outcome of patients with acute strokes (mRS < 1, OR = 1.36, 95% CI: 0.99–1.87). According to the Barthel index (BI > 95) scale, we analyzed five RCT studies to assess the effects of citicoline on the improvement in the daily living function of patients with acute stroke. There was no significant difference in terms of improvement in daily activity function between patients in the experimental group and those in the control group (OR = 1.12, 95% CI:0.81–1.53). 

As for the effect of choline alphoscerate, we evaluated the effect of the compound in comparison to citicoline in five studies with patients who suffered from acute strokes. Two of the five studies included in the present review used the NIHS and mRS to assess neurological function and functional outcomes, while the remaining three studies used the MMSE and Mathew’s scale to measure the primary endpoint. The effects of choline alphoscerate on acute stroke in each of the five studies are summarized below. 

Kamchatnov PR. et al. [41] evaluated the treatment effect of choline alphoscerate (1000 mg IV daily) for 10 days; the 5th and 7th days of the post-treatment follow-up period showed no significant differences between the experimental and control groups. In contrast, 41.6% of the experimental patients showed significant improvement in their neurological function on the 21st day of the post-treatment period, as compared to 18.6% in the control group (*p* < 0.05) based on the NIHSS score. Using the Barthel index, the authors assessed the degree of disability on the 21st day and found that the experimental patients (17.8%) were significantly more independent in everyday life as compared to those in the control group (*p* < 0.05) [41]. Their results indicated that choline alphoscerate significantly improved the outcome of patients who had acute ischemic strokes [41]. 

A randomized trial conducted by Vinogradov OI. et al. [38] (2013) evaluated the effects of choline alphoscerate (1000 mg parenterally and 1200 mg orally) on patients with acute ischemic stroke (a total of 60 patients: 30 in the experimental group and 30 in the control group). The treatment was started 12 h after the onset of the stroke and continued parenterally for 10 days, then orally for 20 days with 400 mg 3 times per day. The investigators assessed the treatment’s effect on neurological function and functional recovery using the NIHSS and mRS. As a result, 50.8% of the experimental patients had significantly improved their neurological function compared to 36.8% of the control group’s patients after 30 days (*p* < 0.05). The data on the evaluation of neurological function using the NIHSS for all patients at baseline (TO) and after 30 days (T1) are summarized below. At baseline and 30 days after treatment, the mean and standard deviation (SD) of the experimental patients at NIHSS were 12.8 + 4.6 and 6.1 + 1.2, respectively, and those in the control group were 12.1 + 5.8 and 9.1 + 1.8. There was a significant difference between the baseline and the results after 30 days for the experimental group, but it was non-significant for the control group. Taking into account the findings of their study, the investigators concluded that choline alphoscerate was effective for both neurological function and functional recovery in patients with acute ischemic stroke [38]. 

Sangiorgi GB et al. [6] (1994) studied the effects of two doses of choline alphoscerate (1000 mg and 1200 mg) on patients with acute strokes. In the first phase, patients were administered 1000 mg of choline alphoscerate intramuscularly daily for 28 days. During the first phase, the authors evaluated the efficacy of choline alphoscerate parenterally using Mathew’s scales based on a minimum score of 35 to ensure that patients had a sufficient level of consciousness to cooperate with the psychometric tests. Mathew’s scale ranges from 0 to 100, and a low score indicates poor consciousness or clinical death (if a score is 0), while a high score indicates a very good level of consciousness. As a result, the investigators determined the scale score at baseline and 28 days after the stroke and calculated the mean difference along with a *p*-value in order to evaluate the functional improvement in the patients after 28 days [6]. 

In the second phase, 1200 mg of choline alphoscerate was administered daily orally for five months, and the efficacy of the treatment was determined by using the MMSE, Critchton Rating Scale (CRS), and Global Deterioration Scale [6]. However, we used only MMSE to determine the effectiveness of the treatment in this review. Consequently, the second phase of the study evaluated the cognitive function of stroke patients using the MMSE, and an optimal score was obtained (MMSE > 23). The remaining two studies also evaluated the effects of choline alphoscerate in patients with acute stroke in both the first and second phases using Mathew’s and MMSE scores [39,40]. The summary of the mean difference trend and the mean value of both Mathew’s scale and the MMSE test score are presented in Table 2 and Table 3 for three studies. 

## 4. Discussion

This study performed a systematic review to determine the efficacy of the choline-containing phospholipids citicoline and choline alphoscerate on neurological function, functional recovery, and independence or improvement in daily living activities in patients with acute ischemic stroke and hemorrhagic stroke. Therefore, we evaluated the effect on neurological function by using the National Institutes of Health Stroke Scale (NIHSS) scores. A total of fifteen items measuring the level of neurological impairment have been incorporated into the tool. The total scores on the NIHSS range from 0 to 42, with higher scores corresponding to more severe cerebral infarcts. Out of the 15 studies included in our systematic review, 9 RCTs were used for the meta-analysis to determine the effect of citicoline on patients with stroke. Following an analysis of our pooled patient data, we found that citicoline was ineffective at improving neurological outcomes at the end of the scheduled follow-up period (NIHSS < 1, OR: 1.05; 95% CI = 0.87–1.27). Moreover, our findings are in agreement with a recent study conducted on 2289 patients with acute ischemic stroke. In that study, there was no significant difference between the group of patients treated with citicoline and the placebo group (OR = 1.09; 95% CI: 0.87–1.36). Dávalos A et al. (2012) reported the neutral effect of citicoline on improving neurological functions [4]. There are also other previous studies that support our findings [5,44].

We evaluated the effect of citicoline on functional recovery using the modified Rankin Score (mRS) in patients with acute ischemic stroke. An mRS score ranges from 0 to 6, where a score of 0 indicates no disability at all and a score of 5 indicates severe disability. Therefore, a patient who scores 5 on the mRS scale is bedridden, incontinent, and requires regular care and close follow-up. According to our meta-analysis using mRS < 1 as the measure of functional outcome, citicoline matched the placebo in terms of its efficacy, and there was no significant difference between the experimental and control groups (mRS < 1, OR = 1.36; 95% CI:0.99–1.87). In other words, our results showed that citicoline does not add any clinical benefit to the outcome of patients with acute stroke regarding their functional impairment. Our results are consistent with those of Zafonte RD et al. [36] and Dávalos A et al. [4], who reported that non-significant improvements in function between the citicoline and placebo groups were observed after the end of the scheduled follow-up days.

We also assessed the effect of citicoline on the reduction in dependency in patients with acute ischemic stroke using the Barthel index (BI) score. In general, the Barthel Index (BI) is used to assess the ability of stroke patients to perform activities of daily living. BI scale scores range from 0 to 100; the lowest score (0) indicates a complete dependency on assistance for activities of daily living, while the highest score (100) indicates independence. Our pooled patient data analysis found that citicoline did not reduce dependency or improve daily living activity function in patients with acute ischemic stroke (BI > 95, OR = 1.12; 95%CI: 0.81–1.53). Our finding is in agreement with the study conducted by Dávalos A et al. [4], who reported citicoline treatment was ineffective in improving daily living activities of acute stroke patients based on BI scale scores (BI > 95, OR = 0.95, 85% CI: 0.77–1.17). The results of two more RCT studies conducted in the United States of America (USA), in which citicoline therapy for acute stroke patients failed to improve activities of daily living [5,32], support our study results. In the present study, we performed a meta-analysis and evaluated the effect of citicoline on functional outcomes in patients with hemorrhagic stroke using the modified Rankin Scale (mRS). Our analysis of pooled patient data showed that citicoline was not found to be effective in improving the functional recovery of patients with hemorrhagic stroke (mRS < 1, OR = 1.75, 95% CI: 0.00–964.00).

Moreover, we systematically analyzed five studies to evaluate the effectiveness of choline alphoscerate in patients with acute strokes. As stated earlier, two of the five studies included in the review utilized the NIHS and mRS to assess neurological function and functional outcomes. The remaining three utilized the MMSE and Mathew’s scale to measure the primary as well as secondary outcomes. In general, the five studies that we evaluated measured the efficacy of choline alphoscerate and reported continuous outcomes. Each study assessed the efficacy of the drug in two phases and reported the results in terms of mean and standard deviation. These studies considered the mean differences along with the *p*-value to measure the effectiveness of choline alphoscerate in improving the outcomes of patients with acute stroke.

We found that in two studies (Kamchatnov PR. et al. (2012) and Vinogradov OI. et al. (2013)), choline alphoscerate significantly improved neurological function and functional recovery. Other three studies assessed choline alphoscerate’s activity, and the results obtained are summarized in Table 2 and Table 3. Accordingly, the positive trend in the mean difference value indicated that the resultant interest of stroke patients had improved, and the score was at a normal level. The results of our review indicated that treatment with choline alphoscerate significantly improved neurological function and functional outcomes in stroke patients. The findings of this study are in accordance with a previously conducted study [13]. This study suggests that choline alphoscerate may have an effect due to its well-known cognitive effects, which may explain some of the improvement in function observed. It has also been reported in a previous study that choline alphoscerate might play a role in acute cerebrovascular diseases by antagonizing the biochemical–functional deficiency of the cholinergic system in cases of ischemia-induced damage [13]. In fact, it has a beneficial effect on patients with neurodegenerative diseases such as Alzheimer’s disease (AD), and the study reported that it significantly improved cognitive function in patients with mild to moderate conditions of AD [45]. As neuroinflammation plays an important role in Alzheimer’s disease, this may be explained by this compound’s potential anti-inflammatory properties [46]. Therefore, this study suggests that choline alphoscerate’s anti-inflammatory effect may improve functioning not only in Alzheimer’s disease but also in acute strokes.

### Limitations of This Study

In order to perform the pooled patient data analysis for the effect of choline alphoscerate on neurological function, functional recovery, and independence or improvement in daily living activity outcomes in patients with acute stroke, we did not find in our literature search the relevant studies with binary outcomes. However, we evaluated the effect of choline alphoscerate using a continuous outcome by calculating the mean difference along with the *p*-value to assess its effectiveness in improving the outcome. Another limitation was that, due to the limited number of RCTs included in the meta-analysis of this systematic review, we were unable to perform meta-regressions or publication bias assessments [47,48]. The small sample size and missing information in some eligible studies prevented us from conducting subgroup analyses to determine the effect of differences by gender, comorbidities, and other relevant factors. Despite the limitations, this study discusses the treatment effects of choline alphoscerate and citicoline in acute stroke patients in comparative ways. 

## 5. Conclusions

Our analysis of pooled patient data suggests that citicoline is not effective in improving neurological function, functional recovery, and independence or improvement in daily living activities in patients with acute stroke. There are also a number of RCT studies that support our findings, and the authors conclude that citicoline does not improve clinical outcomes in patients with acute stroke. Additionally, according to our findings, citicoline treatment does not show evidence of improving functional and neurological outcomes in patients with hemorrhagic stroke who received the drug. Choline alphoscerate, on the other hand, is effective in the improvement in neurological function, functional recovery, and positive outcomes in terms of everyday living activities in patients following an acute stroke. In order to confirm our findings, it is important that future studies make use of a pooled analysis that estimates the odds ratio (OR) for the effect of choline alphoscerate on patients with acute strokes.

## Figures and Tables

**Table 1:** Characteristics of included studies.

Study (Year) Ref.	Study Design	Disease	Patients	Dose Daily	Route of Administration	Duration	Efficacy Measured
Clark WM. et al. (1999) [30]	RCT	Acute stroke	394	500 mg	PO	6 weeks	NIHSS, BI, mRS
Tazaki Y. et al. (1988) [31]	RCT	Acute stroke	267	1000 mg	IV	14 days	mRS
Dávalos A. et al. (2012) [4]	RCT	Acute stroke	2298	1000 mg	IV, PO	6 weeks	NIHSS, BI, mRS
Clark WM. et al. (1997) [32]	RCT	Acute stroke	259	500 mg 1000 mg 2000 mg	PO	6 weeks	NIHSS, BI, mRS, MMSE
Warach S. et al. (2000) [33]	RCT	Acute stroke	81	500 mg	PO	6 weeks	NIHSS and mRS
Agarwal A. et al. (2022) [34]	RCT	Acute stroke	99	1000 mg	IV, PO	6 weeks	NIHSS, BI, mRS
Clark WM. et al. (2001) [5]	RCT	Acute stroke	899	1000 mg	PO	6 weeks	NIHSS, BI, mRS
León-Jiménez C. et al. (2010) [35]	Case-control	Acute stroke	173	1000 mg 500 mg	PO	5–7 weeks	mRS
Zafonte RD. et al. (2012) [36]	RCT	Hemorrhagic stroke	1213	2000 mg	PO	12 weeks	GCS. Processing Speed Index
Secades JJ. et al. (2006) [37]	RCT	Hemorrhagic stroke	38	1000 mg	IV	2 weeks	NIHSS and mRS
Vinogradov OI. et al. (2013) [38]	RCT	Acute stroke	60	1000 mg 1200 mg	IV, PO	30 days	NIHSS and mRS
Sangiorgi GB. et al. (1994) [6]	Uncontrolled trial	Acute stroke	2044	1000 mg 1200 mg	IV, PO	6 months	Mathew’s Scale MMSE, GDS, CGRS
Aguglia E. et al. (1993) [39]	Uncontrolled trial	Acute stroke	425	1000 mg 1200 mg	IM, PO	6 months	Mathew’s Scale MMSE, GDS, CGRS
Tomasina C. et al. (1991) [40]	Uncontrolled trial	Acute stroke	15	1000 mg 1200 mg	IM, PO	6 months	Mathew’s Scale MMSE, GDS, CGRS
Kamchatnov PR. et al. (2012) [41]	RCT	Acute stroke	95	1000 mg	IV	10 days	NIHSS, BI, mRS

**Table 2:** Comparison of the Mathew’s scale scores at baseline and after the first month of treatment with choline alphoscerate for patients with stroke.

Study	Baseline Mean	First Month Mean	Mean Difference	*p*-Value
Sangiorgi GB. et al. (1994) [6]	58.7	74.6	15.90	<0.001
Aguglia E. et al. (1993) [39]	62.02	73.53	11.51	<0.001
Tomasina C. et al. (1991) [40]	61.55	73.40	11.85	<0.03

**Table 3:** A comparison of Mini-Mental State Examination (MMSE) scores between the second and sixth months after the scheduled follow-up period of treatment with choline alphoscerate for patients with stroke.

Study	Second Month Mean	Sixth Month Mean	Mean Difference	*p*-Value
Sangiorgi GB. et al. (1994) [6]	21	24.3	3.30	<0.001
Aguglia E. et al. (1993) [39]	21.55	24.19	2.64	<0.001
Tomasina C. et al. (1991) [40]	22.13	25.5	3.37	<0.05

## Data Availability

All extracted data are available upon request from the corresponding author.

## Supplementary Materials

The following supporting information can be downloaded at: https://www.mdpi.com/article/10.3390/jcm12082875/s1, Table S1: Literature Search strategies and result for PubMed. Table S2: Appraisal of risk of bias of the included randomized controlled trails (RCT) using Cochrane risk of bias tool. Table S3: Quality measures of nonrandomized control trials using Newcastle-Ottawa-Scale.
