# Supplementary material for: Choline-Containing Phospholipids in Stroke Treatment: A Systematic Review and Meta-Analysis
Source: J Clin Med. 2023 Apr 14;12(8):2875. doi: 10.3390/jcm12082875 (PMC10143951; doi:10.3390/jcm12082875)
Supplement: Supplementary file 1 [file jcm-12-02875-s001.zip › jcm-2259703-supplementary.pdf]

Supplementary File

Supplementary Table S1. Literature Search strategies and result for PubMed.

Database	Search key words and combinations	Result
PubMed	((("citicoline"[Title/Abstract] OR "cytidine diphosphate choline"[Title/Abstract] OR "CDP-choline"[Title/Abstract] OR "cytidine 5 diphosphocholine"[Title/Abstract] OR "cyticholine"[Title/Abstract]) AND "acute ischaemic stroke"[Title/Abstract]) OR "acute ischemic stroke"[Title/Abstract] OR "acute ischaemic"[Title/Abstract] OR "acute ischemic"[Title/Abstract] OR "cerebrovascular diseases"[Title/Abstract] OR "brain ischemia"[Title/Abstract] OR "brain ischaemia"[Title/Abstract] OR "cerebrovascular disorders"[Title/Abstract]) AND ((ffrft[Filter]) AND (fha[Filter]) AND (fft[Filter]))	15,746
	(((("Citicoline"[Title/Abstract] OR "cytidine diphosphate choline"[Title/Abstract] OR "CDP-choline"[Title/Abstract] OR "cytidine 5 diphosphocholine"[Title/Abstract] OR "cyticholine"[Title/Abstract]) AND "acute ischaemic stroke"[Title/Abstract]) OR "acute ischemic stroke"[Title/Abstract] OR "acute ischaemic"[Title/Abstract] OR "acute ischemic"[Title/Abstract] OR "cerebrovascular diseases"[Title/Abstract] OR "brain ischemia"[Title/Abstract] OR "brain ischaemia"[Title/Abstract] OR "cerebrovascular disorders"[Title/Abstract]) AND "humans"[Title/Abstract]) AND ((ffrft[Filter]) AND (fha[Filter]) AND (fft[Filter]))	253
	((("Citicoline"[Title/Abstract] OR "cytidine diphosphate choline"[Title/Abstract] OR "CDP-choline"[Title/Abstract] OR "cytidine 5 diphosphocholine"[Title/Abstract] OR "cyticholine"[Title/Abstract]) AND "hemorrhagic stroke"[Title/Abstract]) OR "brain haemorrhage"[Title/Abstract] OR "cerebrovascular accident"[Title/Abstract] OR "intracerebral haemorrhage"[Title/Abstract] OR "subarachnoid haemorrhage"[Title/Abstract] OR "cerebral haemorrhage"[Title/Abstract] OR "cerebrovascular insult"[Title/Abstract]) AND ((ffrft[Filter]) AND (fha[Filter]) AND (fft[Filter]))	4189
	(((("Citicoline"[Title/Abstract] OR "cytidine diphosphate choline"[Title/Abstract] OR "CDP-choline"[Title/Abstract] OR "cytidine 5 diphosphocholine"[Title/Abstract] OR "cyticholine"[Title/Abstract]) AND "hemorrhagic stroke"[Title/Abstract]) OR "brain haemorrhage"[Title/Abstract] OR "cerebrovascular accident"[Title/Abstract] OR "intracerebral haemorrhage"[Title/Abstract] OR "subarachnoid haemorrhage"[Title/Abstract] OR "cerebral haemorrhage"[Title/Abstract] OR "cerebrovascular insult"[Title/Abstract]) AND "humans"[Title/Abstract]) AND ((ffrft[Filter]) AND (fha[Filter]) AND (fft[Filter]))	40
	((("l alpha glycerylphosphorylcholine"[Title/Abstract] OR "alpha-GPC"[Title/Abstract] OR "choline alphoscerate"[Title/Abstract] OR "Cereton"[Title/Abstract] OR "alpha-Glycerylphosphorylcholine"[Title/Abstract] OR "alpha-glyceryl-phosphorylcholine"[Title/Abstract] OR "a-GPC"[Title/Abstract]) AND "acute ischaemic stroke"[Title/Abstract]) OR "acute ischemic stroke"[Title/Abstract] OR "acute ischaemic"[Title/Abstract] OR "acute ischemic"[Title/Abstract] OR "cerebrovascular diseases"[Title/Abstract] OR "brain ischemia"[Title/Abstract] OR "brain ischaemia"[Title/Abstract] OR "cerebrovascular disorders"[Title/Abstract]) AND ((ffrft[Filter]) AND (fha[Filter]) AND (fft[Filter]))	1679

	(((("l alpha glycerylphosphorylcholine"[Title/Abstract] OR "alpha-GPC"[Title/Abstract] OR "choline alphoscerate"[Title/Abstract] OR "Cereton"[Title/Abstract] OR "alpha-Glycerylphosphorylcholine"[Title/Abstract] OR "alpha-glyceryl-phosphorylcholine"[Title/Abstract] OR "a-GPC"[Title/Abstract]) AND "acute ischaemic stroke"[Title/Abstract]) OR "acute ischemic stroke"[Title/Abstract] OR "acute ischaemic"[Title/Abstract] OR "acute ischemic"[Title/Abstract] OR "cerebrovascular diseases"[Title/Abstract] OR "brain ischemia"[Title/Abstract] OR "brain ischaemia"[Title/Abstract] OR "cerebrovascular disorders"[Title/Abstract]) AND "humans"[Title/Abstract]) AND ((ffrft[Filter]) AND (fha[Filter]) AND (fft[Filter]))	127
	((("l alpha glycerylphosphorylcholine"[Title/Abstract] OR "alpha-GPC"[Title/Abstract] OR "choline alphoscerate"[Title/Abstract] OR "Cereton"[Title/Abstract] OR "alpha-Glycerylphosphorylcholine"[Title/Abstract] OR "alpha-glyceryl-phosphorylcholine"[Title/Abstract] OR "a-GPC"[Title/Abstract]) AND "hemorrhagic stroke"[Title/Abstract]) OR "brain haemorrhage"[Title/Abstract] OR "cerebrovascular accident"[Title/Abstract] OR "intracerebral haemorrhage"[Title/Abstract] OR "subarachnoid haemorrhage"[Title/Abstract] OR "cerebral haemorrhage"[Title/Abstract] OR "cerebrovascular insult"[Title/Abstract]) AND ((ffrft[Filter]) AND (fha[Filter]) AND (fft[Filter]))	897
	(((("alpha-GPC"[Title/Abstract] OR "choline alphoscerate"[Title/Abstract] OR "Cereton"[Title/Abstract] OR "alpha-Glycerylphosphorylcholine"[Title/Abstract] OR "alpha-glyceryl-phosphorylcholine"[Title/Abstract] OR "a-GPC"[Title/Abstract]) AND "hemorrhagic stroke"[Title/Abstract]) OR "brain haemorrhage"[Title/Abstract] OR "cerebrovascular accident"[Title/Abstract] OR "intracerebral haemorrhage"[Title/Abstract] OR "subarachnoid haemorrhage"[Title/Abstract] OR "cerebral haemorrhage"[Title/Abstract] OR "cerebrovascular insult"[Title/Abstract]) AND "humans"[Title/Abstract]) AND ((ffrft[Filter]) AND (fha[Filter]) AND (fft[Filter]))	18

Supplementary Table S2. Appraisal of risk of bias of the included randomized controlled trails (RCT) using Cochrane risk of bias tool.

Trial	Method of randomization and concealment of allocation	Blinding of participants, personnel	Blinding of outcome assessors	Incomplete outcome data	Selective outcome reporting	Classification
Clark WM. et al (1999)[24]	Yes	Yes	Yes	No	Yes	Low
Tazaki Y. et al (1988)[25]	Yes	Yes	Yes	No	Yes	Low
Clark WM. et al (1997)[26]	Yes	Yes	Yes	No	Yes	Low
Warach S. et al (2000)[27]	Yes	Yes	Yes	No	Yes	Low
Agarwal A. et al (2022)[28]	Yes	Yes	Yes	No	Yes	Low
Clark WM. et al(2001)[5]	Yes	Yes	Yes	No	Yes	Low
Dávalos A. et al (2012)[4]	Yes	Yes	Yes	No	Yes	Low
Zafonte RD. et al (2012)[30]	Yes	Yes	Yes	No	Yes	Low
Secades JJ. et al (2006)[31]	Yes	Yes	Yes	No	Yes	Low
Vinogradov OI. et al (2013)[32]	Yes	Unclear	Unclear	No	Yes	Moderate
Kamchatnov PR.et al (2012)[35]	Yes	Unclear	Unclear	No	Yes	Moderate

Supplementary Table S3. Quality measures of nonrandomized control trials using Newcastle-Ottawa-Scale.

**Study**	**Selection**				**Comparability**		**Outcome**			**Total**
	1	2	3	4	5	6	7	8	9	Quality score
León-Jiménez C. et al (2010)[29]	*	*	*	*	*	*	*	*	*	**9**
Sangiorgi GB. Et al (1994)[6]	*	*	*	*		*	*	*	*	**8**
Aguglia E. et al (1993)[33]	*	*	*	*			*	*	*	**7**
Tomasina C.et al (1991)[34]	*	*	*	*			*	*	*	**7**

A study may receive a maximum of one star for each numbered item within the category of Selection and Outcome. For Comparability, a maximum of two stars may be awarded. **For the observational studies, selection: (1) representativeness of the exposed cohort, (2) selection of the non-exposed cohort, (3) ascertainment of exposure, (4) demonstration that outcome of interest was not present at start of study. Comparability: (5) study control for age and sex, (6) study control for additional factors. Outcome: (7) assessment of outcome, (8) follow-up long enough for outcomes to occur (9) adequacy of follow up. **For case-control studies**, selection: (1) case definition adequate, (2) representativeness of cases, (3) selection of controls, (4) definition of controls. Comparability:(5) study controls for age and sex, (6) study controls for additional factors. Outcome: (7) ascertainment of exposure, (8) same method of ascertainment for cases and controls, (9) non-response rate.*
